# Die Another Day

**DOI:** 10.5811/westjem.2015.1.24516

**Published:** 2015-02-26

**Authors:** Pablo Aguilera, Oscar Navea, Felipe Arqueros, Mel Herbert

**Affiliations:** * Pontificia Universidad Catolica de Chile, Department of Emergency Medicine, Santiago, Chile; † Keck School of Medicine, University of Southern California, Los Angeles, California

A 22-year-old healthy male university student presented to the emergency department (ED) complaining of syncope. He had five episodes of loss of consciousness from 10 to 40 seconds in length, with loss of postural tone and full recovery without intervention in the last month. Witnesses to these events denied tonic-clonic activity, and he had no sphincter tone loss. On the index ED visit the patient was in good condition, without distress. Primary survey was normal, he denied chest pain, dyspnea or headache. He had no history of tobacco or illicit drug use, and was a moderate social drinker. Secondary survey was unremarkable.

An electrocardiogram (ECG) was ordered during the ED visit. During the performance of the test the patient collapsed, and the ECG showed he had degenerated from sinus rhythm to ventricular fibrillation (VF) (Figure 1). Cardiopulmonary resuscitation with chest compressions were commenced immediately. He was defibrillated with 200 J biphasic shock, which returned him to sinus rhythm. He recovered consciousness after the shock and remained hemodynamically stable. A second ECG post defibrillation showed sinus rhythm with a right bundle branch block pattern and ST segment elevation in V1–V4 leads, compatible with a type 1 Brugada syndrome. The patient was admitted to the coronary critical care unit, and was evaluated by the electrophysiologist who confirmed the diagnosis. The patient had an automated implantable cardiac defibrillator inserted as an inpatient. He subsequently had no more episodes of VF in hospital and was discharged.

## DISCUSSION

**Figure 2:**
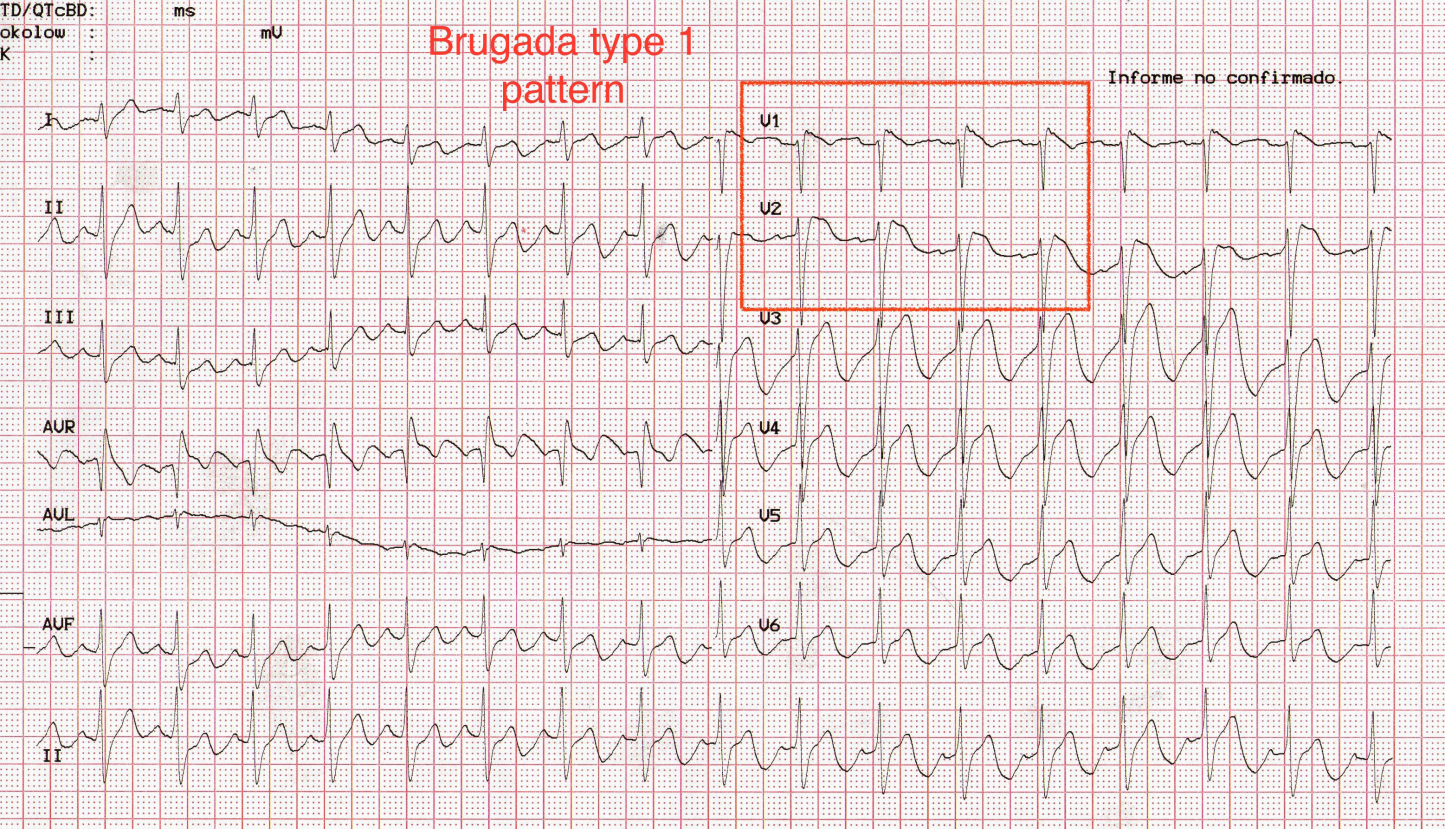
Twelve-lead electrocardiogram after successful defibrillation. Notice the Brugada type I pattern on the anterior leads.

Brugada syndrome is a well-described cause of sudden death in young patients.^1^ The resting ECG of these patients classically shows a characteristic incomplete or complete right bundle branch block pattern with ST-segment elevation in leads V1–V2 (Figure 2).

This is a unique case because an episode of spontaneous ventricular fibrillation was caught during the performance of a routine screening ECG. The ECG shows the dramatic transition from sinus rhythm to coarse ventricular fibrillation in a torsade de pointes-like pattern.

Definitive testing for Brugada syndrome should be done in the electrophysiology laboratory. If the diagnosis is confirmed, an automated implantable cardioverter-defibrillator (AICD) should be considered and placed.

This case is a reminder for emergency practitioners to look for the signs of Brugada syndrome on ECG in all patients who present with a history of syncope, since AICD placement is an effective and life-saving treatment. Baseline mortality in patients with Brugada syndrome can be as high as 10% per year, and this is essentially reduced to zero with AICD placement.^2^ In this case the ECG morphology was consistent with a type 1 or coved-type Brugada syndrome. Type 1 Brugada syndrome is the most clinically important as it has the highest rate of spontaneous degeneration to ventricular fibrillation.^3^

## Figures and Tables

**Figure 1:**
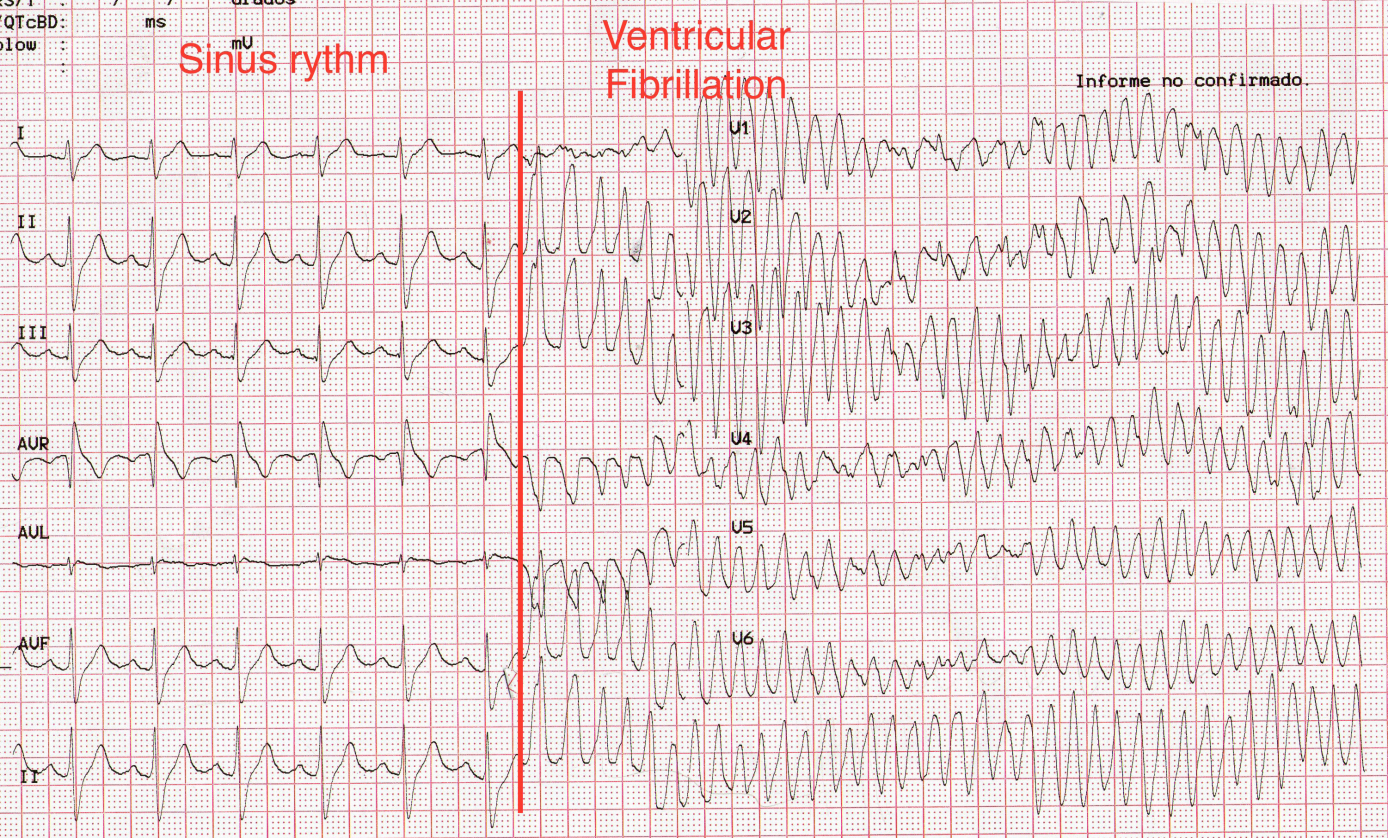
Twelve-lead electrocardiogram showing the degeneration from sinus rhythm to ventricular fibrillation.
